# Human monocytes and macrophages differ in their mechanisms of adaptation to hypoxia

**DOI:** 10.1186/ar4011

**Published:** 2012-08-07

**Authors:** Monique Fangradt, Martin Hahne, Timo Gaber, Cindy Strehl, Roman Rauch, Paula Hoff, Max Löhning, Gerd-Rüdiger Burmester, Frank Buttgereit

**Affiliations:** 1 Department of Rheumatology and Clinical Immunology, Charité University Hospital, Charitéplatz 1, Berlin, 10117 Germany; 2 German Rheumatism Research Center (DRFZ), Charitéplatz 1, Berlin, 10117 Germany; 3 Berlin-Brandenburg School for Regenerative Therapies (BSRT), Foehrer Straße 15, Berlin, 13353 Germany; 4 Berlin-Brandenburg Center for Regenerative Therapies (BCRT), Foehrer Straße 15, Berlin, 13353 Germany

## Abstract

**Introduction:**

Inflammatory arthritis is a progressive disease with chronic inflammation of joints, which is mainly characterized by the infiltration of immune cells and synovial hyperproliferation. Monocytes migrate towards inflamed areas and differentiate into macrophages. In inflamed tissues, much lower oxygen levels (hypoxia) are present in comparison to the peripheral blood. Hence, a metabolic adaptation process must take place. Other studies suggest that Hypoxia Inducible Factor 1-alpha (HIF-1α) may regulate this process, but the mechanism involved for human monocytes is not yet clear. To address this issue, we analyzed the expression and function of HIF-1α in monocytes and macrophages, but also considered alternative pathways involving nuclear factor of kappa light polypeptide gene enhancer in B-cells (NFκB).

**Methods:**

Isolated human CD14^+ ^monocytes were incubated under normoxia and hypoxia conditions with or without phorbol 12-myristate 13-acetate (PMA) stimulation, respectively. Nuclear and cytosolic fractions were prepared in order to detect HIF-1α and NFκB by immunoblot. For the experiments with macrophages, primary human monocytes were differentiated into human monocyte derived macrophages (hMDM) using human macrophage colony-stimulating factor (hM-CSF). The effects of normoxia and hypoxia on gene expression were compared between monocytes and hMDMs using quantitative PCR (quantitative polymerase chain reaction).

**Results:**

We demonstrate, using primary human monocytes and hMDM, that the localization of transcription factor HIF-1α during the differentiation process is shifted from the cytosol (in monocytes) into the nucleus (in macrophages), apparently as an adaptation to a low oxygen environment. For this localization change, protein kinase C alpha/beta 1 (PKC-α/β_1 _) plays an important role. In monocytes, it is NFκB1, and not HIF-1α, which is of central importance for the expression of hypoxia-adjusted genes.

**Conclusions:**

These data demonstrate that during differentiation of monocytes into macrophages, crucial cellular adaptation mechanisms are decisively changed.

## Introduction

Inflammatory arthritis is characterized by infiltration of immune cells and local tissue hypoxia [1-3]. Mature monocytes migrate towards sites of inflammation and infection where they differentiate into inflammatory macrophages or into dendritic cells (DCs) [1,4,5]. Macrophages have the highest phagocytic potential of cells in the area of inflammation. They also present the antigen components previously processed via the major histocompatibility complex II (MHC II) and, via cytokines, attract other macrophages, granulocytes and T-cells into inflamed areas.

Monocytes and macrophages require energy in the form of adenosine triphosphate (ATP) in order to facilitate motion (migration to achieve phagocytosis), activation (signal cascade, initiation, maintenance, cytokine production) and effector functions (phagocytosis, antigen presentation, regulatory functions) [4]. Under aerobic conditions, the energy supply of the cells occurs via oxidative phosphorylation (OxPhos) and glycolysis. In the absence of oxygen (that is, under hypoxic conditions), OxPhos does not occur and only glycolysis remains for ATP production, because this process does not require oxygen [6,7].

Hypoxia occurs in joint inflammation such as during the pathogenesis of rheumatoid arthritis [1,5], fracture hematomas [8], and malignant tumors [9]. Ng *et al. *demonstrated in recent *in vivo *studies that an inverse correlation exists between synovial oxygen partial pressure and inflammatory activity in patients with arthritis: the stronger the inflammation, the more pronounced the local hypoxia. Kennedy *et al. *showed anti-TNF-therapy, widely used to treat RA, increases the oxygen partial pressure in the joint. For the initial inflammatory environment in an early fracture hematoma, the specific cytokine pattern and typical gene signatures in immune cells reflect a situation of local hypoxia [8]. In addition, Vaupel *et al. *showed the important role of hypoxia and hypoxia-inducible factors (HIFs) in tumorigenesis [9].

During monocytopoiesis, monocyte precursor cells in the bone marrow, monocytes in the blood and macrophages in the tissue are subjected to very different oxygen levels. For example, an oxygen partial pressure (pO_2_) of < 10 mmHg (1.3% O_2_) is present in the bone marrow of mice [10]. In contrast, much higher pO_2 _values of 50 to 100 mmHg (6 to 13% O_2_) in the peripheral blood and of 20 to 50 mmHg (approximately 2.5 to 6.0% O_2_) in healthy tissue were measured [11]. Furthermore, in inflamed areas macrophages face pathophysiological hypoxia. In these regions, the oxygen content is lower than in healthy tissues because of imbalance between provision and consumption of oxygen. Disturbed blood circulation and inflammatory swellings resulting in a lengthening of the diffusion distance decrease the oxygen supply whereas the influx of metabolically active immune cells strongly increases the oxygen consumption [12]. For these reasons, cells are forced to adapt immediately to the reduced pO_2 _levels when entering these low oxygenated areas. The mechanism of this adaptation and the temporal relationship of this response to activation and migration are not yet fully understood.

Rapid adaptation of monocytes to hypoxia may involve HIF or other factors. In other cells (for example, T-cells), it is known that the transcription factor HIF-1 under hypoxic conditions is translocated into the nucleus and binds to promoter regions of target genes to enable the necessary adaptation and maintenance of basic functions like motion, activation and effector cell function [12,13]. However, there are divergent views on the expression and function of HIF in primary human monocytes. Neither HIF-1α, HIF-2α nor HIF-3α were found by Elbarghati *et al. *in primary monocytes after incubation under hypoxia for 24 h. The authors suspected that the α-subunit of HIF is not expressed, because the peripheral blood as a place of residence of circulating monocytes is characterized physiologically by a high pO_2 _[11,14]. However, it should be noted, that monocytes have to adapt to lower oxygen levels immediately once they start the process of being attracted to the vessel's wall, migrating into the inflamed tissue and starting to differentiate into macrophages. CXCR4 transcript levels have been shown to increase in monocytes facing hypoxia, which suggests HIF is crucially involved in regulating the trafficking [15]. Furthermore, in myeloid cell lines like THP-1 cells incubated under hypoxia, HIF-1α was detectable [16]. There have also been reports that nuclear factor of kappa light polypeptide gene enhancer in B-cells (NFκB), a transcription factor also regulated by hypoxia, is involved in the adaptation of primary human monocytes to hypoxia [17].

Here we have examined how human monocytes adapt to hypoxic conditions during their differentiation into macrophages. We focused on the analysis of expression and function of HIF-1, but also considered alternative pathways involving NFκB.

## Materials and methods

### Antibodies and reagents

PMA, macrophage colony stimulating factor 1 (hM-CSF), and GÖ 6976 were purchased from Sigma Aldrich Chemie GmbH (Hamburg, Germany), ImmunoTools (Friesoythe, Germany), Merck KgaA (Darmstadt, Germany). For toll like receptor (TLR) stimulation, hTLR ligand Set II was bought from Apotech (Epalinges, Switzerland). For immunoblotting, mouse monoclonal anti-HIF-1α antibody was bought from BD Transduction Laboratories (Heidelberg, Germany); mouse anti-β-actin antibody was purchased from Sigma-Aldrich (Hamburg, Germany); goat polyclonal anti-HIF-2α, goat polyclonal anti-Lamin B and mouse anti-Jun-B antibody were bought from Santa Cruz Biotechnology (Heidelberg, Germany); mouse monoclonal anti-NFκB p100/p52, anti-NFκB p105/p50, anti-NFκB p65, anti-c-Rel, anti-c-Fos, anti-c-Jun antibody were bought from Cell Signaling (Frankfurt/Main, Germany); anti-mouse IgG HRP and anti-goat IgG HRP were bought from Promega (Mannheim, Germany).

### Monocyte isolation, culture and macrophage differentiation

Human peripheral blood (buffy coats) was obtained from healthy donors (DRK-Blutspendedienst Ost gemeinnützige GmbH, Berlin/Brandenburg/Sachsen, Germany, with approval from the Charité Ethics Review Board). Peripheral blood mononuclear cells from these buffy coats were then immediately isolated by density gradient centrifugation using Ficoll-Paque™ Plus technique (Amersham Bioscience AB, Freiburg, Germany). To ensure a stable experimental setup and comparable starting conditions, CD14^+ ^monocytes were enriched up to 99% purity and > 95% viability (data not shown) by MACS using anti-human CD14 conjugated magnetic beads (Miltenyi Biotec, Bergisch Gladbach, Germany) and then immediately used for the experiments. Monocytes were cultured at 4 × 10^6^cells/ml in RPMI 1640 supplemented with 10% volume/volume percent (v/v) heat-inactivated FCS (Sigma-Aldrich, Hamburg, Germany), 100 units/ml penicillin G, 100 μg/ml streptomycin (both PAA Laboratories, Pasching, Austria), and 50 μM 2-ME (Sigma-Aldrich, Hamburg, Germany). Monocytes were incubated with 25 nM hM-CSF for 7 days to differentiate into monocyte derived macrophages (hMDM).

### Culture of cell lines

THP-1, HL-60, and U937 cells (American Type Culture Collection) were cultured in Roswell Park Memorial Institute media (RPMI) 1640 supplemented with 10% (v/v) heat-inactivated FCS, 100 units/ml penicillin G, 100 μg/ml streptomycin, and 50 μM 2-ME. Human microvascular endothelial cells (HMEC-1) were purchased from the Center of Disease Control (Atlanta) and were cultivated in endothelial basal medium (PAA Laboratories GmbH, Pasching, Austria) supplemented with 5% (v/v) heat-inactivated FCS (Sigma-Aldrich, Hamburg, Germany), 100 units/ml penicillin G, 100 μg/ml streptomycin (both PAA Laboratories GmbH, Pasching, Austria), 1% (v/v) 200 mM L-glutamine, 0,01% (v/v) endothelial growth factor (EGF) (100 μg/ml), 0,2% (v/v) hydrocortisone (380 μM) (both Sigma) and grown in 0.2% gelatine-coated 75 cm^2 ^culture flasks or 24-well plates, respectively.

### Induction of hypoxia and stimulation

Cells were incubated in a humidified hypoxic chamber (Binder, Tuttlingen, Germany) at 5% CO_2 _level and less than 1% O_2 _balanced with N_2_. Normoxic controls were incubated at 5% CO_2 _in a humidified atmosphere with 18% O_2_. Stimulation was done with PMA 10 ng/ml (16 nM) (Sigma Aldrich Chemie GmbH, Hamburg, Germany). For kinetic analyzes under hypoxia, monocytes were incubated in a water-jacket chamber sealed with a Clark-type oxygen electrode (Strathkelvin Instruments, North Lanarkshire, Scotland) as described previously [12].

### RNA isolation and quantitative real-time PCR (qPCR) of selected genes

After cell lysis, total RNA was extracted (RNeasy Mini Kit; Qiagen, Hilden, Germany) and the quality was assessed on a Bioanalyzer (Agilent). The cDNA was synthesized by reverse transcription using TaqMan^® ^Reverse Transcription Reagents (Applied Biosystems, Darmstadt, Germany). qPCR was carried out using the LightCycler^® ^Fast Start DNA Master SYBR^® ^Green I Kit (Roche, Mannheim, Germany). Data were normalized to the expression of β-actin (ACTB). All primers used were obtained from TIB Molbiol (Berlin, Germany): β-actin (*ACTB*), *gACAggATgCAgAAggAgATCACT, TgATCCACATCTgCTggAAggT*; hypoxia-inducible factor 1, alpha subunit (*HIF1A*), *CCATTAgAAAgCAgTTCCgC, TgggTAggAgATggAgATgC; *lactate dehydrogenase A (*LDHA*), *ACCCAgTTTCCACCATgATT, CCCAAAATgCAAggAACACT; *phosphoglycerate kinase 1 (*PGK1*), *ATggATgAggTggTgAAAgC, CAgTgCTCACATggCTgACT; *chemokine-receptor 4 (*CXCR4*), *ggCATgACggACAAgTACAggCT, AAAgTACCAgTTTgCCACggC *(Table 1).

**Table 1:** Primersets used

Gene symbol	Gene name	Gene function	Primerset
***ACTB***	beta-actin	Structural housekeeper	*gACAggATgCAgAAggAgATCACT* *TgATCCACATCTgCTggAAggT*

***HIF1A***	hypoxia-inducible factor 1, alpha subunit	Transcription factor	*CCATTAgAAAgCAgTTCCgC* *TgggTAggAgATggAgATgC*

***LDHA***	lactate dehydrogenase A	glycolysis enzyme	*ACCCAgTTTCCACCATgATT* *CCCAAAATgCAAggAACACT*

***PGK1***	phosphoglycerate kinase 1	glycolysis enzyme	*ATggATgAggTggTgAAAgC* *CAgTgCTCACATggCTgACT*

***CXCR4***	chemokine-receptor 4	chemokine-receptor	*ggCATgACggACAAgTACAggCT* *AAAgTACCAgTTTgCCACggC*

### Immunoblotting

Cell lysis: for whole cell extracts of monocytes, 10^6 ^cells were lysed in 20 μl Laemmli buffer. For the preparation of nuclear/cytoplasmic fraction, 4*10^6 ^cells were lysed using the Nuclear Extract Kit from Active Motif (La Hulpe, Belgium), according to the manufacturer's instructions.

Immunodetection of proteins: 20 μl of whole cell extract or nuclear/cytoplasmic fraction was separated by SDS-PAGE and blotted onto polyvinylidene difluoride membranes (Millipore, Darmstadt, Germany). Blotted proteins were analyzed as indicated and visualized by enzymatic chemiluminescence (Amersham Biosciences, Freiburg Germany).

### Statistical analyzes

Data shown are reported as the mean ± SD of at least six experiments. Differences between normally distributed groups were compared using the Student's *t*-test and in non-normally distributed groups with the Mann-Whitney *U*-test for independent groups and with the Wilcoxon *t*-test for dependent samples.

## Results

### HIF-1α is stabilised under hypoxia in human monocytes but remains in the cytoplasm

**Figure 1:**
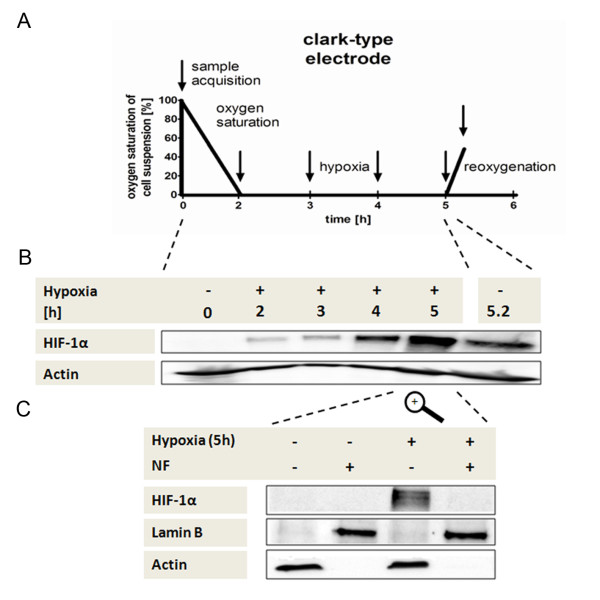
**Hypoxia-inducible factor alpha (HIF-1α) is stabilized under hypoxia but remains in the cytoplasm**. (**A**) Scheme of sample acquisition for kinetic analyzes of monocytes incubated in a water-jacket chamber sealed with a Clark-type oxygen electrode, which facilitates the constant monitoring of oxygen saturation during the experimental setup (sample acquisition is indicated by arrows). (**B**) Detection of HIF-1α and, for normalization purposes, β-actin in monocyte whole cell protein samples acquired as shown in (A) by immunoblot. Under hypoxia, monocytes stabilize HIF-1α in a time-dependent manner. (**C**) Detection of HIF-1α, β-actin and for normalization purposes, the nuclear protein Lamin B, in monocyte nuclear fraction (NF+) and cytosolic (NF-) cell fractions using immunoblot. HIF-1α was exclusively detected in the cytoplasm in unstimulated monocytes incubated under hypoxic conditions (*n *= 6).

In order to investigate first the stabilisation of HIF-1α as a function of pO_2 _values and duration of incubation, MACS-isolated CD14^+ ^monocytes were incubated in a Clark-type electrode for 5 h (Figure 1A) with a subsequent reoxygenation time of 12 minutes. Immunoblot analyzes revealed that monocyte stabilisation of HIF-1α begins when hypoxia (pO_2 _< 2%) commences. With increasing duration of hypoxia, the accumulation of HIF-1α increased (Figure 1B). There was marked accumulation of HIF-1α during incubation under hypoxia. As expected, reoxygenation caused an immediate degradation of HIF-1α (Figure 1B).

Next, we analyzed the cytosolic and nuclear fractions after 5 h incubation, in order to define the exact location of HIF-1α. HIF-1α was found exclusively in the cytoplasm and not in the nuclear fraction of hypoxic monocytes (Figure 1C).

### TLR stimulation does not affect HIF-1α localization

**Figure 2:**
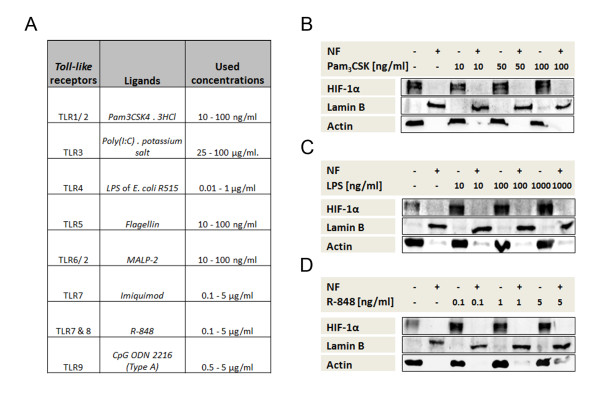
**Toll-like receptor (TLR) stimulation does not affect hypoxia-inducible factor alpha (HIF-1α) localization**. (**A**) Table of TLRs and their corresponding ligands used in our experiments within the given concentration range. (**B**-**D**) Detection of HIF-1α, Lamin B and β-actin in monocyte nuclear (NF+) and cytosolic (NF-) cell fractions using immunoblot. Monocyte protein lysates were acquired after TLR stimulation and incubation for 5 h under hypoxia using (**B**) TLR1/2 stimulation by Pam_3_CSK_4 _3HCl, (**C**) TLR4 stimulation by LPS or (**D**) TLR7/8 stimulation by R-848.

Following these observations, we investigated whether concurrent TLR stimulation of human hypoxic monocytes is needed for translocation of HIF-1α into the nucleus. We incubated the cells for 5 h under hypoxia, with concurrent stimulation of TLR1-9 using a range of ligands (Figure 2A). TLR stimulation under hypoxic conditions did not lead to translocation of HIF-1α into the nucleus, regardless of the ligand and concentration used. Representative experimental results are shown in Figure 2B-D, obtained with Pam_3_CSK_4_3HCl (toll-like receptor (TLR)-1/2 stimulation), lipopolysaccharide LPS (TLR4 stimulation) and R-848 (TLR7/8 stimulation). Under all hypoxic conditions tested, HIF-1α was detectable exclusively in the cytosol fraction of primary human monocytes.

### PKC-α/β_1 _is essential for HIF-1α translocation

**Figure 3:**
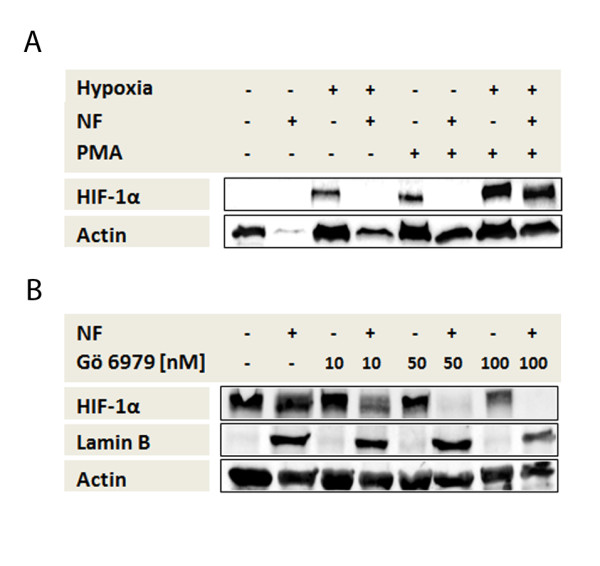
**Protein kinase (PKC)-α/β_1 _is essential for hypoxia-inducible factor alpha (HIF-1α) translocation**. (**A+B**) Detection of HIF-1α, Lamin B and β-actin in nuclear (NF+) and cytosolic (NF-) cell fractions of primary human monocytes using immunoblot. (A) Monocyte protein lysates were acquired after PMA stimulation and incubation for 5 h under hypoxia and under normoxia as indicated. (B) Monocyte protein lysates were acquired after PMA stimulation following PKC-α/β_1 _inhibition using Gö6976 as indicated and subsequent incubation for 5 h under hypoxia. (**A**, **B**) PMA stimulation for 5 h under hypoxia leads to nuclear translocation of HIF-1α, and specific inhibition of PKC-α/β_1 _by Gö6976 prevents translocation of HIF-1α.

We examined whether stimulation with PMA leads to translocation of HIF-1α into the nucleus. PMA is usually applied to differentiate monocytes over a short time to a macrophage-like phenotype. HIF-1α cannot be found in unstimulated monocytes when incubated under normoxia, as shown by immunoblot analyzes (Figure 3A). However, if the cells were stimulated with PMA for 5 h under normoxia, a weak HIF-1α signal in the cytosol fraction was detectable. Although HIF-1α was detectable under hypoxia in unstimulated monocytes exclusively in the cytoplasm, in hypoxic PMA-stimulated monocytes it was detectable not only in the cytoplasm, but also in the nucleus. The signal strength of HIF-1α seen in hypoxic PMA-stimulated cells was stronger than in hypoxic unstimulated monocytes.

Since PMA is known to be a PKC activator, we incubated monocytes for 5 h under hypoxia stimulated with PMA, with concurrent addition of the PKC-α/β_1_- inhibitor, Gö6976, at increasing concentrations. Figure 3B shows that the inhibitor at a concentration of 50 nM reduced the accumulation of HIF-1α in the nucleus. With a Gö6976 concentration of 100 nM, HIF-1α was no longer detectable in the nucleus. These data demonstrate that PKC-α/β_1 _is essential for the transport of HIF-1α from the cytoplasm into the nucleus.

### Monocyte differentiation to macrophages leads to HIF-1α translocation

We considered whether differentiation of human monocytes into macrophages (hMDMs) using hM-CSF also caused hypoxia-induced translocation of HIF-1α into the nucleus. The macrophage nuclear fraction was identified using the location of Lamin B; the location of actin was not used as this may be found in the nuclear fraction of macrophages due to alterations in the cytoskeleton of macrophages after stimulation, as described by Hartwig and Janmey [18].

**Figure 4:**
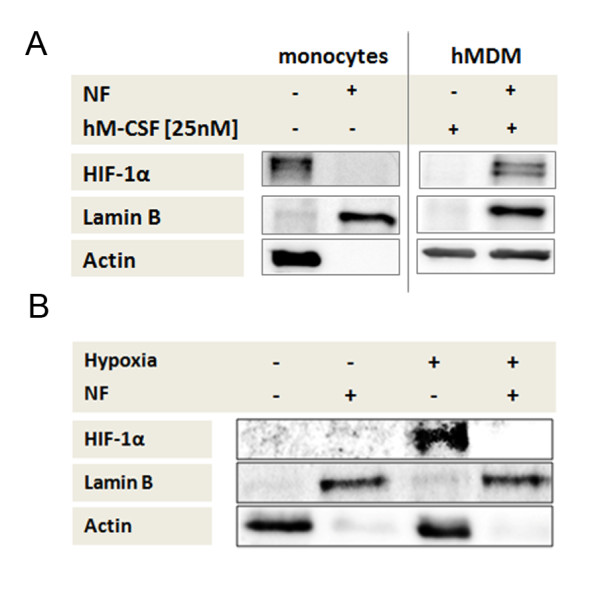
**Hypoxia-inducible factor alpha (HIF-1α) translocation after differentiation of monocytes to macrophages and co-culture with endothelial cells**. (**A**, **B**) Detection of HIF-1α, Lamin B and β-actin in monocyte or hMDM nuclear (NF+) and cytosolic (NF-) cell fractions using immunoblot. (**A**) Protein lysates were acquired after incubation of human monocytes and hMDM for 5 h under hypoxia. Differentiation of monocytes to macrophages leads to HIF-1α translocation into the nucleus. (**B**) Monocyte protein lysates were acquired after incubation of human monocytes on human endothelial cells (HMEC-1, human microvascular endothelial cells) coated well plates for 5 h under normoxia and under hypoxia. Co-culture of monocytes with endothelial cells is not sufficient for the translocation of HIF-1α.

Incubation of monocytes with 25 nM hM-CSF over 7 days led to differentiation into hMDM. After incubation of monocytes and hMDMs for 5 h under hypoxia, HIF-1α in the monocytes could only be detected in the cytosolic compartment, while in hMDMs HIF-1α was seen to reside in the nuclear extract (Figure 4A).

We also considered whether any possible prior adhesion of human monocytes to endothelial cells, as an initial step of migration, could initiate the translocation of HIF-1α into the nucleus. Human monocytes were incubated for 5 h in plates with wells coated with human microvascular endothelial cells (HMEC-1), under hypoxia and under normoxia. The cellular localization of HIF-1α was investigated. We observed that co-culture with endothelial cells of this type did not induce accumulation of HIF-1α in the nucleus of the monocytes. HIF-1α was found exclusively in the cytosol fraction of monocytes, as assessed by immunoblot (Figure 4B); HIF-1α was not detectable under normoxia. Taken together, these findings suggest that adhesion of monocyte to the vascular wall is not sufficient for translocation of HIF-1α to the nucleus.

### Hypoxia-induced gene expression of human monocytes versus hMDMs

Next we compared the expression of selected hypoxia-induced genes in human monocytes and hMDMs. We examined genes that have been identified as typical HIF-1 target genes such as the glycolysis enzymes *LDHA *and *PGK1*, and the chemokine receptor *CXCR4*.

**Figure 5:**
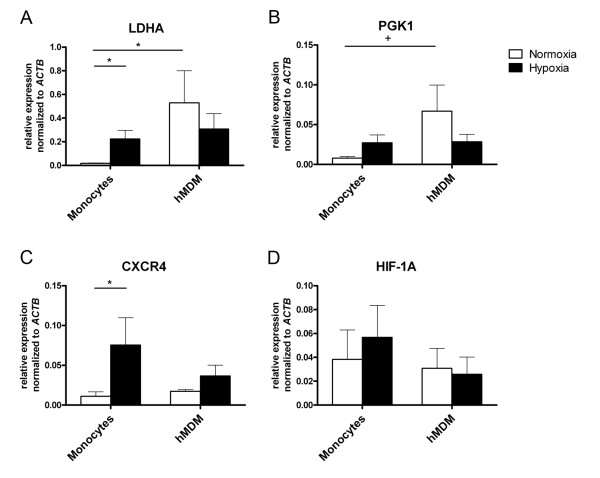
**Monocytes, in contrast to human monocyte derived macrophages (hMDMs), show hypoxia-induced gene expression**. Real-time PCR analysis of mRNA expression levels of the glycolytic enzymes *LDHA *(**A**) and *PGK1 *(**B**), the hypoxia inducible factor (HIF)-1 target gene *CXCR4 *(**C**) and the transcription factor *HIF1A *(**D**) in monocytes and hMDM that were incubated under normoxia (white bars) and under hypoxia (black bars), respectively (*n *= 6). The mRNA expression level of ß-actin (ACTB) was used for normalization. ΔCt = Ct_(GOI) _- Ct_(ACTB)_. Values are means ± SD. ^+^*P *< 0.1;* *P *< 0.05; (Wilcoxon *t*-test).

In monocytes incubated under hypoxic conditions - in contrast to normoxia - genes for *LDHA *and *CXCR4 *were significantly (*P *< 0.05) upregulated, although HIF-1α is not present in the nucleus; the gene for *PGK1 *showed increased expression but this did not reach statistical significance (Figure 5A-C). *HIF1A *as a target gene of HIF-1 itself is regulated at the protein level and therefore showed no measurable induction (Figure 5D). Macrophages under normoxia showed higher expression of the genes *LDHA *(1.7-fold) and *PGK1 *(2.3-fold) than monocytes (Figure 5A and 5B). There was no significant difference in the expression of these genes under hypoxia versus normoxia, presumably because metabolism had already been switched from oxidative phosphorylation/respiration to anaerobic glycolysis during differentiation.

Similar findings were observed for *CXCR4 *(Figure 5C), where hypoxic incubation did not lead to a significant increase of *CXCR4 *expression in hMDMs. Also in hMDMs, hypoxia did not induce any measurable upregulation of *HIF1A *(Figure 5D).

### Incubation of monocytes under hypoxia leads to translocation of transcription factor NFκB1 into the nucleus

As the expression of HIF target genes (for example, *LDHA*, *CXCR4) *in monocytes was induced by hypoxia although HIF-1α was not present in the nucleus, we considered other transcription factors that could be involved.

**Figure 6:**
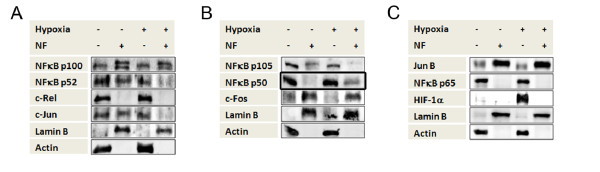
**Incubation of monocytes under hypoxia leads to translocation of transcription factor, nuclear factor of kappa light polypeptide gene enhancer in B-cells (NFκB1), into the nucleus**. Detection of hypoxia-inducible factor alpha **(**HIF-1α), Lamin B and β-actin in nuclear (NF+) and cytosolic (NF-) cell fractions of primary human monocytes using immunoblot. (**A**-**C**) Monocyte protein lysates were acquired after incubation for 5 h under hypoxia and under normoxia as indicated. The cellular localization of transcription factors NFκB p100/p52, c-Rel, and c-Jun (Figure 6A), NFκBp105/p50 and c-Fos (Figure 6B), and Jun B and NFκB p65 (Figure 6C) were determined by immunoblot. NFκBp105 (the inactive form of NFκB1) remains in the cytoplasm, while the active form NFκBp50 is translocated into the nucleus under hypoxic conditions (Figure 6B). Other factors showed either no change in their localization or, as for c-Jun and NFκBp52, were no longer translocated into the nucleus under hypoxic incubation.

We examined the cellular localization of transcription factors NFκB p100/p52, c-Rel, and c-Jun (Figure 6A), NFκBp105/p50 and c-Fos (Figure 6B), and Jun B and NFκB p65 (Figure 6C) in monocytes that were incubated for 5 h under hypoxia. Although other transcription factors (for example, c-Fos and NFκB p65) did not change their localization when incubated under hypoxic versus normoxic conditions, NFκBp105 (the inactive form of NFκB1) remained in the cytoplasm, while the active form NFκBp50 was translocated into the nucleus. The active form of NFκB2 (NFκBp52) and c-Jun could be seen in the nucleus under normoxic, but not under hypoxic conditions.

### THP-1, HL-60, and U937 cells express HIF-1α in the cell nucleus under hypoxic and normoxic conditions

Myeloid cell lines are often used as an experimental model for primary human monocytes. We considered in which cellular compartment HIF-1α could be found in unstimulated and PMA-stimulated myeloid cell lines (THP-1, HL-60, and U937) under hypoxic conditions. We identified HIF-1α in the nucleus both under normoxic and hypoxic conditions with or without PMA stimulation (See Figure S1 A-C in Additional file 1). We conclude that in this regard, the cell lines clearly differ from primary human monocytes and behave like hMDMs. This is of concern as these cell lines, but not human monocytes, are routinely used for research on bioenergetic issues (for example, adaptation to hypoxia).

## Discussion

Circulating blood monocytes face oxygen concentrations of more than 40 mmHg, which fuel oxidative phosphorylation. However, upon migration to inflamed joints, monocytes encounter hypoxic conditions and must adapt immediately to the reduced pO_2_. For several different cell types, it has been shown that the transcription factor HIF-1 under hypoxic conditions is translocated into the nucleus where it binds to promoter regions of target genes. This enables cells to adapt and maintain their basic and specific functions [19,20]. Elbarghati *et al. *reported recently that primary human macrophages but not monocytes rapidly up-regulate HIF-1α and HIF-2α proteins upon exposure to hypoxia, with translocation of these proteins into the nucleus [14].

We demonstrate here that the transcription factor HIF-1α also accumulates in quiescent human monocytes under hypoxia, but is present solely in the cytosol. For this reason, we postulate that it cannot be responsible for the transcriptional induction of typical hypoxia target genes in the nucleus. It is not clear why monocytes differ in this regard from many other cell types, where HIF-1α under hypoxia is translocated into the nucleus [21-25]. One possibility is that the HIF-induced adaptation mechanism in monocytes is not necessary because of the plentiful oxygen supply present in peripheral blood. The stabilisation of HIF-1α in the cytosol under hypoxic conditions may, therefore, reflect a pre-active state that becomes active when the cells start their migration into low oxygen tissue areas. However, it should be noted that we also studied quiescent and PHA-stimulated peripheral human blood CD3^+^CD4^+ ^T-cells, which also circulate in oxygen-rich blood. In contrast to monocytes, hypoxic conditions induced HIF-1α in these cells, with translocation into the nucleus as shown by immunoblotting [12]. From this observation, we suggest that the HIF-1α regulation mechanism may be a feature of the evolutionary younger cells of the adaptive immune system, but not of evolutionary older cells of the innate immune system, such as monocytes.

The apparent lack of involvement of HIF-1α in regulating expression of hypoxia-induced genes in monocytes suggests that other transcription factors mediate this effect. In the literature, it has been reported that adaptive responses to hypoxia are regulated by several transcription factors, including HIF-1, HIF-2, ETS-1, cAMP response element binding protein, activator protein-1 and nuclear factor-κB [26-33]. Hence, we examined various possible transcription factors and found that the active form of NFκB1, NFκBp50, is translocated into the nucleus of the human monocytes as a reaction towards a pO_2 _of < 2%. In good agreement with this observation, Battaglia *et al. *have previously shown DNA binding of NFκBp50 under hypoxia in primary human monocytes by means of a supershift analysis [17]. Furthermore, Oliver *et al. *have described the selective activation of the canonical NF-κB pathway via p65 by intermittent and sustained hypoxia in HeLa cells [34]. The non-canonical NF-κB pathway via p52 is not impacted by hypoxia. Our results are consistent with these findings as we show, to our knowledge for the first time in primary monocytes, that p50 is part of the canonical pathway induced by sustained hypoxia.

We therefore suggest that NFκB1 serves as a key regulator enabling the immediate adaptation of monocytes to hypoxia during migration from blood into the tissue environment. We suggest that, during the differentiation process of human monocytes into macrophages, the more potent and possibly more robust HIF-1 system is activated. The HIF-1 system may be needed for the rapid adaptation to varying oxygen concentrations, which is of essential functional importance for long-living tissue macrophages. Indeed, we demonstrate here that the stimulation of the monocytes with PMA (which although non-physiological is usually applied to differentiate monocytes over a short time to a macrophage-like phenotype) and the more physiological induction of monocyte differentiation by means of M-CSF cause the translocation of HIF-1α into the nucleus of long-living tissue macrophages. The presence of HIF-1α in the nucleus of macrophages or hMDMs under hypoxia has already been verified by other groups [14,35-37]. HIF-1α was also detectable in the nucleus of different myeloid cell lines (THP-1, HL-60, and U937) under hypoxic conditions. Although often used as experimental models of monocytes, these cell line cells are highly proliferative and malignant cells with numerous differences from macrophages, hMDMs, and monocytes. With regard to the HIF-1 pathways, these cell lines behave like macrophages or hMDMs, but not like monocytes. This should be considered when using these cell lines as models to analyze the bioenergetic functions of monocytes and/or macrophages in inflammatory arthritis.

Our observation that both PMA stimulation and M-CSF-induced differentiation of monocytes into macrophages causes the translocation of HIF-1α into the nucleus prompted a search for potential regulators. Since PKC-α/β_1 _is strongly activated by PMA stimulation, we hypothesized that this protein kinase enzyme could play a key role. Indeed, our experiments demonstrated that the inhibition of PKC by Gö6976 leads to abrogation of HIF-1α translocation into the nucleus. Our observation is in agreement with data provided by Chang and Beezhold who have proved the existence of both prevailing PKC-isoforms α and β in primary human monocytes [38]. Lin *et al. *recently showed Gö6976-mediated inhibition of PKC-α and membrane translocation during differentiation into MDMs, and consequently diminished differentiation of MDMs [39]. It should be noted, however, that the exact mechanism of the PKC-mediated transport of HIF-1α into the nucleus is currently still unclear.

It was interesting to realize that HIF-1α is not shifted into the nucleus if human monocytes are incubated under hypoxia with concurrent TLR stimulation. Furthermore, contact of monocytes with endothelial cells (that is,. the initial migration step and therefore the beginning of the differentiation) is not sufficient to induce HIF-translocation. Taken together, these data clearly demonstrate that neither the contact of monocytes with antigen in the hypoxic areas nor the contact of monocytes with endothelial cells causes the translocation of HIF-1α into the nucleus; rather, it appears to be the differentiation process *per se *that activates the HIF-1 system.

In agreement with Bosco *et al. *[40], we showed that hypoxia strengthens the genetic expression of the glycolysis enzyme *LDHA*. HIF-1α is not present in the nucleus under these conditions so we infer this effect to be mediated by NFκBp50. However, macrophages demonstrate significantly higher expressions of the gene *LDHA *(and another enzyme involved in glycolysis, *PGK1*) under normoxia than monocytes. In addition, the expression of these genes is not increased by incubating macrophages under hypoxic conditions (*LDHA P *= 0.39, PGK *P *= 0.26). A constitutional PKC over-expression constantly inducing the HIF-1 system may be a possible explanation of these observations [41].

Interestingly, the observations made for the glycolysis genes differ from those for the chemokine receptor *CXCR4*. It should be noted that the expression of this chemokine receptor is oxygen-dependent. Therefore, the chemotactic behaviour of monocytes can be adapted to variable oxygen conditions. Bosco *et al. *described a genetic induction of *CXCR4 *in a transcriptome characterisation of monocytes incubated under hypoxia [40]. We also found the *CXCR4 *gene in monocytes to be significantly induced under hypoxia. However, in contrast to glycolysis genes, the *CXCR4 *gene expression under normoxia in hMDMs is not more pronounced than in monocytes. Although *CXCR4 *expression increased in hMDMs with hypoxia, this increase was not significant (*P *= 0.17). A significant increase in the genetic expression for *CXCR4 *under hypoxia in hMDMs has been described by Fang *et al. *[42]. Schioppa *et al. *showed that in human monocytes and human MDMs, hypoxia induced expression of *CXCR4 *at the protein level [15]. The authors interpret the navigation process hypoxia/HIF-1/CXCR4 as an important mechanism for the regulation of cell migration into hypoxic tissues or for the retention of cells in hypoxic tissues. This is in line with our finding of hypoxia/HIF-1/CXCR4 for hMDMs but, due to the absence of HIF-1α in the nucleus of monocytes, as hypoxia/NFκBp50/CXCR4 for monocytes.

## Conclusions

In summary, the localization of the transcription factor HIF-1α is shifted during the differentiation process from the cytosol (in monocytes) to the nucleus (in macrophages), apparently as a PKC-α/β_1_- mediated adaptation to a low oxygen environment. In monocytes, it is NFkB1, and not HIF-1α, that is of central importance for the expression of hypoxia-adjusted genes. These data demonstrate that (i) during differentiation crucial cellular adaptation mechanisms are decisively changed and (ii) bioenergetic aspects are of crucial importance for the understanding of underlying pathophysiological processes in inflammatory arthritis.

## Abbreviations

ACTB: ß-actin; ATP: adenosine triphosphate; c-AMP: cyclic adenosine monophosphate; CD: cluster of differentiation; cDN: complementary deoxyribonucleic acid; CO_2_: carbon dioxide; c_t_: threshold cycle; CXCR: C-X-C motif chemokine receptor; DC: dendritic cell; DRK: Deutsches Rotes Kreuz (German Red Cross); EGF: endothelial growth factor; FCS: fetal calf serum; GÖ 6976: 12-(2-Cyanoethyl)-6,7,12,13-tetrahydro-13-methyl-5-oxo-5H-indolo[2,3-a]pyrrolo[3,4-c]carbazole; HeLa: Henrietta Lacks cell line; HIF: hypoxia-inducible factor; HL-60: human promyelocytic leukemia cells; hM-CSF: human macrophage colony-stimulating factor; hMDM: human monocyte derived macrophages; HMEC-1: human microvascular endothelial cell line; HRP: horseradish peroxidase; IgG: immunoglobulin G; LDHA: lactate dehydrogenase A; LPS: lipopolysaccharide; MACS: magnetic activated cell sorting; 2-ME: ß-mercaptoethanol; MHC: major histocompatibility complex; mmHg: millimeter of mercury; N_2_: molecular nitrogen; NF: nuclear fraction; NFκB: nuclear factor of kappa light polypeptide gene enhancer in B-cells; O_2_: molecular oxygen; OxPhos: oxidative phosphorylation; Pam_3_CSK_4_3HCl: Pam3Cys-Ser-(Lys)4-Trihydrochloride; PCR: polymerase chain reaction; PGK1: phosphoglycerate kinase 1; PHA: phytohemagglutinin; PKC: protein kinase C; PMA: phorbol 12-myristate 13-acetate; pO_2_: oxygen partial pressure; qPCR: quantitative polymerase chain reaction; R-848: resiquimod; RA: rheumatoid arthritis; RNA: ribonucleic acid; RPMI 1640: Roswell Park Memorial Institute media 1640; SD: standard deviation; SDS-PAGE: sodium dodecyl sulfate polyacrylamide gel electrophoresis; THP-1: human acute monocytic leukemia cell line; TLR: toll like receptor; TNF: tumor necrosis factor; U-937: human histiocytic leukemia cell line; v/v: volume/volume percent.

## Competing interests

The authors declare no competing financial or non-financial interests.

## Authors' contributions

All authors have made substantial contributions to conception and design of the study, or to acquisition of data and the analysis and interpretation. All authors read and commented on the draft versions of the manuscript and have given final approval of the version to be published.

## Supplementary Material

Additional file 1 **Figure S1 A-C. Myeloid cell lines (THP-1, HL-60, and U937) express HIF-1α in the cell nucleus**. THP-1 cells (**A**), HL-60 cells (**B**) and U937 cells (**C**) express HIF-1α in the nucleus under normoxia and hypoxia, with or without PMA stimulation (5 h). THP-1 and U937 show similar expression of HIF-1α under normoxia and hypoxia (**A**,**C**), whereas HL-60 cells demonstrate increased expression of hypoxia-inducible factor (HIF)-1α under hypoxia (**B**). All cell lines show a higher expression of HIF-1α in the presence of PMA (**A**-**C**). Detection of HIF-1α, Lamin B and β-actin in nuclear (NF+) and cytosolic (NF-) cell fractions of myeloid cell lines using immunoblot. (**A**-**C**) Protein lysates were acquired after incubation for 5 h under hypoxia and under normoxia as indicated. Click here for file
