# Supplementary figures and images for: Human monocytes and macrophages differ in their mechanisms of adaptation to hypoxia
Source: Arthritis Res Ther. 2012 Aug 7;14(4):R181. doi: 10.1186/ar4011 (PMC3580576; doi:10.1186/ar4011)

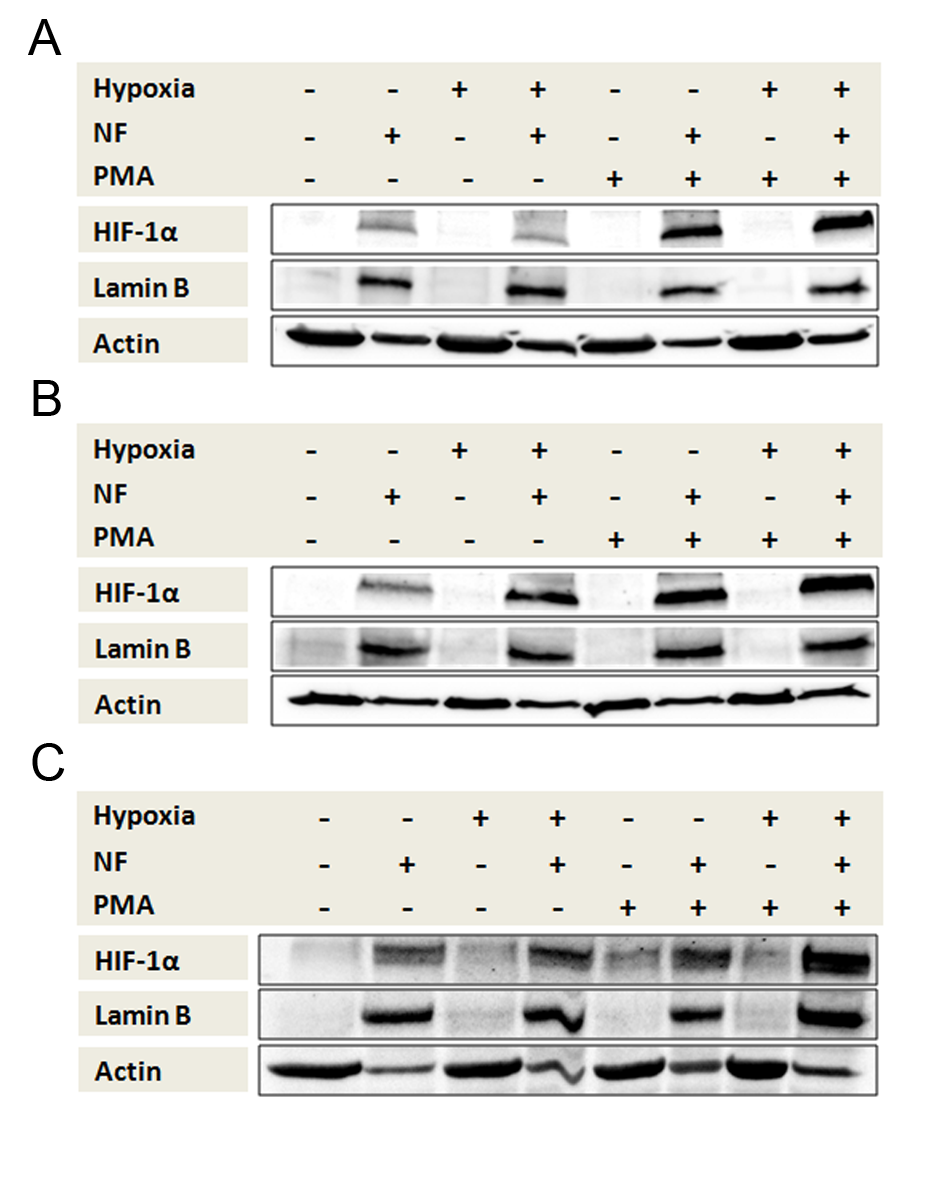

Supplement: Additional file 1 — Figure S1 A-C. Myeloid cell lines (THP-1, HL-60, and U937) express HIF-1α in the cell nucleus. THP-1 cells (A), HL-60 cells (B) and U937 cells (C) express HIF-1α in the nucleus under normoxia and hypoxia, with or without PMA stimulation (5 h). THP-1 and U937 show similar expression of HIF-1α under normoxia and hypoxia (A,C), whereas HL-60 cells demonstrate increased expression of hypoxia-inducible factor (HIF)-1α under hypoxia (B). All cell lines show a higher expression of HIF-1α in the presence of PMA (A-C). Detection of HIF-1α, Lamin B and β-actin in nuclear (NF+) and cytosolic (NF-) cell fractions of myeloid cell lines using immunoblot. (A-C) Protein lysates were acquired after incubation for 5 h under hypoxia and under normoxia as indicated. [file ar4011-S1.TIFF]
